# The benefits and disappointments following clitoral reconstruction after female genital cutting: A qualitative interview study from Sweden

**DOI:** 10.1371/journal.pone.0254855

**Published:** 2021-07-21

**Authors:** Malin Jordal, Hannes Sigurjonsson, Gabriele Griffin, Anna Wahlberg

**Affiliations:** 1 Center for Gender Research, Uppsala University, Centrum för genusvetenskap, Uppsala, Sweden; 2 Department of Health and Caring Sciences, University of Gävle, Gävle, Sweden; 3 Department of Plastic and Reconstructive Surgery, Karolinska University Hospital, Stockholm, Sweden; 4 Department of Medicine, Karolinska Institutet, Solna, Sweden; 5 International Maternal and Child Health, Department of Women’s and Children’s Health, Uppsala University, Uppsala, Sweden; Erasmus Medical Center, NETHERLANDS

## Abstract

Female genital cutting or mutilation refers to the cutting of girls’ external genitalia. Due to migration from contexts where female genital cutting is common, it is estimated that around 38 000 cut women and girls live in Sweden. Clitoral reconstruction, a relatively new form of surgical healthcare offered to women with female genital cutting, was established in Sweden in 2014. This surgery aims at restoring clitoral function and anatomy, but there is yet a dearth of evidence demonstrating the effects of the surgery. The aim of this study was to explore how women undergoing clitoral reconstruction in Sweden between 2016 and 2019 experienced the surgical process and its aftereffects from a physical, sexual and psychosocial perspective. Eighteen women who had undergone clitoral reconstruction at a university hospital in Sweden agreed to participate in the study. The women were interviewed using semi-structured interviews, which were recorded, transcribed and analysed using thematic analysis. The results, based on self-categorization and labelling theory, demonstrated both benefits and disappointments following the surgery. Several women reported positive outcomes in terms of sexual, psychosocial and aesthetic terms. They experienced reduced genital pain, improvements in their sex lives, and a sense of feeling more empowered and at ease in their bodies. Yet, some women reported aesthetic, functional and process-related disappointment related to clitoral reconstruction. Nonetheless, the women expressed gratitude for the possibility of undergoing the surgery. In conclusion, the women reported that they experienced physical, sexual and psychosocial benefits of the surgery.

## Introduction

Female genital cutting (FGC) refers to the cutting of girls’ external genitalia, often without their consent, with no documented medical benefits [1]. FGC may take different forms, whereof some involve cutting the external clitoris [2]. The World Health Organization (WHO) [2] typically divides FGC into four types. Type I involves partial or total removal of the clitoris and/or the prepuce (clitorectomy), Type II partial or total removal of the clitoris and the labia minora, with or without excision of the labia majora (excision), and Type III a narrowing of the vaginal orifice with creation of a covering seal (infibulation), with or without excision of the external parts of the clitoris. Type IV refers to all other harmful procedures to the female genitalia for non-medical purposes, such as pricking, piercing, incising, and scraping. It is estimated that around 200 million women and girls all over the world have gone through some form of FGC [3]. Due to migration, an estimated half a million cut women and girls now live in Europe [4]. In Sweden, more than 38 000 cut women and girls have undergone FGC, the majority from Somalia [5].

### FGC and the effect on health, sexuality and bodily identity

Cut women and girls have a higher risk of genitourinary, obstetric and psychosexual health problems (anxiety, depression, dyspareunia, lack of sexual desire and reduced sexual satisfaction) than non-cut women [6–9]. Yet, the psychosexual health consequences are complex and difficult to measure. Thus recordings of the effects of FGC on sexual function are not coherent [9–12].

While FGC is considered a sign of beauty and womanhood in one context, it may signify violence and repressive traditionalism in another [13, 14]. Thus, migration from one context to another is likely to affect cut women’s perceptions of their bodies, self and identity [15]. Consequently, FGC-related problems may be context specific. Studies have demonstrated that psychosexual complaints are higher in low-prevalence countries than in high-prevalence ones [16, 17]. One explanation might be that FGC is normalized in high-prevalence countries so that its effects are taken for granted whilst this might not be the case in low-prevalence countries. Another may be that women are enabled to speak out about these effects in one context but not in another. Yet another explanation might be that exposure to educational material about the negative effects of FGC on sexual function makes women experience problems they would otherwise not recognize if not exposed to such messages [16]. On the other hand, it may be that the educational material enables women already experiencing sexual dysfunction to make sense of and report sexual dysfunction.

### Healthcare for women with FGC

Migrants’ equitable access to healthcare has received increased attention in recent years. In many European countries this has translated into efforts to improve the healthcare for women with FGC [17, 18]. Healthcare for these women generally involves defibulation, removal of cysts and keloids, psychosexual counselling, and in the recent years, clitoral reconstruction (CR) [17, 19]. The rationale for CR is to restore clitoral function and anatomy [20]. Women asking for surgery generally report that they want to improve their sexual function and restore their self-image as ‘whole’ women [21–23].

CR was developed by Pierre Foldès in the French and West African context [20]. In recent years this surgery has become increasingly popular in other European countries [24]. In Sweden, CR was introduced into public healthcare in 2014 [25]. Because CR is part of specialist care in Sweden, women who want it are required to seek referral for the surgery via primary healthcare, often their own gynaecologist or a specialized clinic providing care for FGC-affected women in Stockholm. CR is technically a relatively simple surgical procedure and involves removal and dissection of scar tissue covering the remaining parts of the clitoris [16]. The technique of CR used in Sweden is a modified version of Foldès’ [20] method; it involves incision of the skin over the clitoral shaft stump. Dissection is proceeded cranially to the clitoral stump, preserving the dorsal neurovascular bundle. Close to the pubic bone, the suspensory ligament is transected to a degree that it allows sufficient mobilization to an appropriate anatomical position. A neo-glans is created and sutured to the skin with projection after resection of residual scar and fibrotic tissue. In selected cases mucosal skin from the vaginal wall was grafted onto the neo-glans for faster healing and better aesthetical appearance. Concomitant reconstructive surgery (e.g. defibulation, removal of cysts) is done if necessary [26]. Since 2014, over 40 women have undergone surgery at the Karolinska University Hospital in Sweden. CR treatment does not only involve surgery, but also pre-operative psychosexual consultations as a requirement for receiving surgery, a popular model in many public clinics in Europe [24, 27]. All the women undergoing CR are routinely offered psychosexual consultations also after the surgery.

There were two parts to this study. The first part involved interviewing women asking for CR about their motives for and expectations of the surgery. This is reported elsewhere [28]. The second part involved interviewing the same women again after surgery to ask about the perceived aftereffects of CR. This is what this article centres on. The first part of the study demonstrated that the cut women seeking CR in Sweden viewed FGC as a form of patriarchal violence and misogynist oppression, which troubled their sense of self [28]. They wanted to have surgery to improve their sexual capacity, feel whole, symbolically take back power over their own bodies, and reduce physical pain. They had heard about the availability of CR through the radio, TV, newspapers, the Internet, a specialist FGC clinic, their GP or friends [28]. This seems to differ from other countries where CR is promoted through media campaigns and advertisements [16]. In this second part of the study we aim at exploring how the same group of women experienced the surgical process and its aftereffects from a physical, sexual and psychosocial perspective, and thus whether CR leads to the expected benefits.

## Method

### Design, recruitment, data collection and analysis

The study has a qualitative design. This is useful when wanting to explore multifaceted, in-depth perspectives in an under-researched area with few available participants [29]. Participants were recruited purposively with the inclusion criterion of having undergone CR after FGC. The majority of the women had already been interviewed pre-operatively. At the time of the pre-operative interview, all the women had been asked and agreed to participate in a follow-up interview. Of the 17 women interviewed pre-operatively, 15 had undergone CR and were thus eligible for this follow-up study. Of these, 14 could be contacted (one woman could not be contacted, most probably because she had moved abroad) and agreed to be interviewed. In addition, two women who had been interviewed pre-operatively but not been included in the pre-surgery study, and two women who had undergone surgery, but not been interviewed pre-operatively, were asked to participate in the second study. All the women who were reached agreed to participate. In total, 18 women were included in this follow-up study of women having undergone CR.

The data was collected by the first author between 2016 and 2019, approximately one to two years after the CR, utilizing semi-structured interviews. One woman (Anna) was interviewed only six months after surgery as she planned to travel abroad. While the intention was to interview the women around one year post surgery, travels, pregnancies or delay in responding to the interviewer’s text messages or emails sometimes delayed this period. The interviews took place in the participant’s home, in a private room at the hospital premises, at a library, or in a cafe, depending on practicalities and interviewees’ preferences. The interviews focused on the experience of going through CR surgery and whether expectations related to the surgery had been met. In most cases, the interviewer had already established rapport during the first interview. The interviewer attempted to create a trustful environment in all interviews by displaying a non-judgmental and empathetic attitude [30], as well as asking about FGC and CR in a neutral language.

Sixteen interviews were conducted in Swedish, one in English, and one in Somali using a telephone interpreter. Fourteen interviews were conducted face-to-face and four over the telephone. The interviews lasted between 23 and 80 minutes. Sixteen interviews were tape-recorded and later transcribed. In the remaining two, notes were taken because the women did not feel comfortable with being recorded.

The interviewer, who is a female nurse and researcher (postdoc), emphasised that she was not connected to the care institution other than through working with the surgeon on the study design and recruitment, and without investment in whether the surgery was ‘successful’ or not. Nevertheless, it could be sensed that the women sometimes found it difficult to express negative experiences or disappointment with the surgery. To balance out any potential bias, the interviewer made efforts to probe into the ‘less than positive experiences’. When dissatisfaction and uncertainty regarding the aftereffects of the surgery were expressed, the interviewer encouraged the woman to contact the surgeon. In some cases, the interviewer, on the participant’s request, contacted the surgeon to inform him that she wanted contact. This was done without revealing any content from the interview to the surgeon.

The participants were first provided with a letter containing information about the purpose and procedure of the study, that the data would be treated confidentially, and that they had the right to withdraw from the study at any time without an explanation. Before starting the recording, the interviewer went through the information letter orally. The interviewees were also informed that their decision to participate or not would not affect their treatment. Further, they were informed that they did not have to answer any question if feeling uncomfortable. Oral consent was obtained and documented by the interviewer prior to starting the interview. Oral, and not written, consent was chosen so as not to cause unease to the participants with having to sign a written form, something that was approved by the Regional Ethical Review Board in Stockholm, from where the study received ethical approval (2015/1188-31). The content of the interviews has been anonymized for all authors besides the first author, and pseudonyms are used for all participants.

Thematic analysis was undertaken to analyse the data [31]. All the interview transcripts were read closely and repeatedly by the first author, looking for patterns in the responses related to the study aim. Data extracts were selected and coded based on the content, and the codes were organized into themes capturing the substance of the data set. This process was a ‘back and forth’, thus not linear, movement between codes, data extracts, the entire data set and preliminary themes. The final themes were established when the process was considered exhausted and complete, capturing the core message in the data set.

In analysing the data, we proceeded, theoretically, from the assumption that the biological/somatic and the socio-cultural are not distinct but co-constructed [32–35]. Hence, although for analytical purposes we discuss the aftereffects of CR under distinct headings we regard the biological/somatic and the socio-cultural as entangled. As our findings show, issues of the socio-cultural were as significant to our interviewees as biological/somatic ones; indeed, they were interwoven since surgical interventions impacted on the interviewees’ emotions and sense of self as much as on the biological/somatic.

### Research participants

The participants were aged between 21 and 58 years. They had immigrated to Sweden from Somalia (n = 9), Eritrea (n = 3), Gambia [n = 2), Irak (n = 2), Senegal (n = 1), or Sierra Leone (n = 1). They had lived in Sweden between 3 and 29 years, the majority (n = 15) more than 10 years. Many worked in the healthcare sector, mostly as nurses or nurse assistants. Others worked as personal assistants or cleaners, were students or unemployed. One woman worked as an engineer. Four women were married, nine unmarried and five divorced. Several of the divorced and unmarried women had a boyfriend or were engaged at the time of the interview. Eight of the women had children, and one had grandchildren. All the women had residency rights in Sweden. The majority of the women had undergone FGC Type III (n = 10). Yet, only three women were still infibulated at the time of requesting CR surgery, with the remaining ones having been defibulated earlier, at least partly, due to childbirth or for other reasons. Six women had undergone Type II and two women Type I FGC. Table 1 provides an overview of the key characteristics of the participants.

**Table 1:** Characteristics of women having undergone CR in Sweden.

Pseudonym	Age range	Country of origin	Years in Sweden (range)	Current employment	Civil status	FGC Type
Anna	31–40	Eritrea	21–30	Health care	Divorced	Type II
Barni	31–40	Eritrea	21–30	Unemployed	Unmarried	Type II
Helen	31–40	Eritrea	11–20	Health care	Unmarried	Type II
Ami	31–40	Gambia	21–30	Health care	Unmarried	Type II
Fatou	31–40	Gambia	0–10	Unskilled	Married	Type II
Behar	41–50	Irak	11–20	Health care	Married	Type I
Zara	31–40	Irak	11–20	Engineer	Unmarried	Type I
Fanta	41–50	Senegal	21–30	Health care	Married	Type II
Patricia	21–30	Sierra Leone	11–20	Health care	Unmarried	Type II
Aisha	51–60	Somalia	21–30	Unknown	Divorced	Type III
Ayaan	31–40	Somalia	0–10	Health care	Divorced	Type III
Ilham	21–30	Somalia	11–20	Student	Unmarried	Type III
Imtesam	21–30	Somalia	0–10	Student	Unmarried	Type III
Leila	31–40	Somalia	11–20	Health care	Divorced	Type III
Marua	21–30	Somalia	0–10	Health care	Unmarried	Type III
Natalie	21–30	Somalia	11–20	Health care	Unmarried	Type III
Ruquia	31–40	Somalia	11–20	Student	Divorced	Type III
Soheila	31–40	Somalia	21–30	Health care	Married	Type III

## Findings and discussion

The study demonstrated that the majority of the interviewed women were largely satisfied with having undergone CR. Post-surgery many enjoyed sex more, felt less anxious and at ease in their bodies, and experienced reduced physical genital pain compared to pre-surgery. These improvements affected their intimate relations and their overall lives. Yet, not all women were satisfied with all aspects of the surgery; some reported resentment related to the access, process or aftereffects of CR. Nevertheless, the majority expressed gratitude towards the Swedish healthcare system for providing CR free of charge.

### From ‘drastically improved’ to ‘no change at all’: The effects of CR on sexuality

Many of the women seeking CR had hoped, at least partly, that their capacity to feel sexual desire and pleasure would improve [28]. For many this expectation had also been realized, at least to some extent (thirteen women said they had had sex with a partner after the surgery; of these eight said that their sexual lives had improved from ‘a little’ to ‘very much’). Fatou and Patricia were two women who said that their sex lives had improved drastically after CR. Despite having the same sexual partner as before the surgery, they described radical changes in their capacity to feel pleasure and reach orgasm. Whereas before surgery they had mostly endured sex for the sake of their partner despite feeling anxiety, lack of desire, dryness, pain, and never reaching orgasm, they now experienced sexual desire, ability to lubricate easily, and reaching orgasm. Fatou said laughingly: ‘*Before I did it [had sex] for my husband*, *now I do it for myself’*.

Marua was another woman who was satisfied with her post-surgery sex life, although having never had sex prior to surgery due to being infibulated. Undergoing defibulation and CR in two steps as this was recommended by the surgical team, she said that post CR she desired sex to such a degree that her boyfriend called her ‘insatiable’. Yet, it was clitoral stimulation that gave her the most pleasure: ‘*I prefer when he uses his finger or tongue [on the clitoris]*, *then I don’t want him to stop*.*’* The issue of clitoral stimulation and pleasure was also apparent in the women’s accounts of masturbation. Although several women said they were unaccustomed to masturbation, the majority of those who had masturbated described improved clitoral sensation and pleasure. Even if not experiencing improved sex with a partner, Ami described a difference when masturbating: ‘*There is not much difference during sex [with a partner]*, *I can still not relax*, *but there is a difference with myself*.*’* Barni was another woman who said that her clitoris had become more sensitive after CR: ‘*I can feel that it [the clitoris] is much more sensitive now and it is easier to reach orgasm*, *it doesn’t need as much stimuli as before*’.

While the present study did not systematically measure the difference in clitoral sensitivity before and after CR, it is notable that many women reported a newfound ability to reach orgasm through clitoral stimulation. The importance of the clitoris for women’s sexual pleasure and orgasm has been widely recognized [36]. Yet, in heterosexual encounters the coital imperative and penetrative norm is still strong. This affects how women’s sexual pleasure and orgasm through clitoral stimulation is perceived [37]. Studies attempting to measure the effect of either FGC or CR on sexual capacity do not often differentiate between vaginal and clitoral sex [10, 38], which may be an indication of the assumed coital imperative.

When asked what aspects of CR the women thought contributed to their improved ability to feel sexual pleasure, they said that it was likely a combination of both physical and psychosocial factors. Some pointed towards the physical restoration of clitoral tissue, which was assumed to have direct importance for sexual pleasure. Fanta said: ‘*I didn’t know if it was true that you who have a clitoris enjoyed more*, *but now I can see that there is a difference’*. Others, such as Patricia, highlighted the psychological aspect of CR including the ability to feel less anxious and thus more relaxed, whole, beautiful, and bodily secure in intimate relations: *‘I also believe it [improved sex] is due to feeling more secure’*. Yet, the women found it difficult to single out one reason for their improved sex lives. This may not be surprising as sexual pleasure and capacity is complex; as Johnsdotter [39] writes there are no sexual experiences unmediated by the context, culture and our relations to our bodies.

Some women described their improved capacity to enjoy sex as related to reduced physical pain during sexual intercourse. Aisha recounted that she normally experienced pain when having sex, which she endured to keep her partner satisfied and thus the relationship intact. Post-surgery she reported that the pain had disappeared:

*Before I had pain and it was difficult and I didn’t want to show it [to my partner]. Rejection would imply lack of success [for him] and that felt difficult… But now it feels good and it doesn’t hurt*.

Yet, not all women reported improvements in their sex lives or capacity to reach orgasm, although none of the women experienced a worsening. Those who did not experience any difference related this to their ‘new’ clitoris not being much more sensitive than before surgery, or that they were as unable to relax with a partner as before the surgery. Soheila was one such woman. Yet, she said that she did not prioritize sex at this point in life as she was pregnant and had a small child at home. While partly resenting the lack of improvement in their sex lives, none of the women regretted having had surgery, since they had wanted to undergo CR for other reasons too. Soheila said: *‘It is ok that the sex is not better*, *I did it to take back the power over my body*.*’*

### The psychosocial and aesthetic effects of CR–quitting the category of ‘cut’

One motivation to have CR had been to restore their genitals aesthetically, which the women considered ‘damaged’ due to FGC [28]. Accordingly, several women said that they were satisfied with the appearance of their post-CR genitals as they looked more ‘normal’ than before. This did not necessarily mean that they thought their genitals resembled an uncut vagina. Several women said that they knew that CR could not make their genitals look ‘100% uncut’. Aisha compared her post-surgery genitals to how they looked before: ‘*Who do I compare myself with*? *I do not compare myself with one who haven’t done it [FGC]*, *but with what I had*. *And there is a big difference between what I had and what I have today*.*’*

The issue of ‘normality’ is complex, and is as McDougall [40] suggests culturally, not biologically, determined. Aisha’s statement above demonstrates that her comparison of her genitals related both to its previous appearance and the ideal of uncut genitals, and that she perceived her post surgery genitals to be closer to uncut, or ‘normal’, genitals than before the surgery. Holliday [41] discusses the increased visibility of the female genitals in contemporary culture, which she, despite this visibility’s association with the porn industry, also relates to achievements in feminist struggles. Thanks to this, she writes, the vagina is ‘no longer shameful and hidden but rather a vagina to be proud of, looked at, explored, examined and appreciated’ (34:199). This also means that female genitals have become something in need of management, care and attention [41]. Thinking that their genitals had been ‘damaged’ due to FGC, the women in the present study felt the need for such management. Yet, in the case of the interviewed women, the need to manage their genitals was not reduced to a matter of ‘beauty’. Fusaschi [42] writes that ‘in all societies, practices that interfere with bodies are aimed at (dis)adapting them to gendered cultural norms and making them more socially (un)appropriate’. One might argue that FGC itself is such an interference. However, in contemporary Sweden it signifies not only ‘damaged’ genitals, but a practice belonging to ‘the Other’ [43]. Undergoing CR was therefore partly of symbolic importance. This might signify a way of adapting the body to what is considered culturally appropriate in one specific place, at a specific time. According to Foucault [44] there is a ‘hierarchizing structure of differences’ that categorizes groups and individuals differently and produces social in- or/and exclusions. The self- and other categorization of ‘women with FGC’ produces a kind of exclusion in a society where FGC is seen as divergent. CR can thus give women an ability to approximate to existing gendered and cultural norms by ‘quitting the category of cut’. A relief of no longer considering themselves to belong to the category of ‘cut’ made it easier for some women to talk about FGC, as they thought that FGC was no longer something that was applicable to them. Barni said: ‘*It [CR] is something I am very happy to have done*, *and I think there is a difference*, *like*, *it is easier for me to talk about the FGC now*, *than it has ever been before*.’

No longer marked by the ‘sign of FGC’ also meant being able to ‘pass as normal’ [45]. This involved the ability not to tell intimate partners about their FGC, which provided a sense of control. Barni said: ‘*Now I can choose whether I want to tell [about FGC to a partner]*, *I*, *like*, *have that control*. *That means a lot*.’ Yet such ‘change of categories’ [44] did not necessarily have to be to ‘uncut’; for some women it sufficed to modify their FGC. Marua and Imtesam had both been infibulated at the time of seeking surgery and were able to convince their new partners that they had only undergone a minor form of FGC, which was considered ok. Marua said: ‘*He [boyfriend] asked about FGC*, *and I said that my mother only did a little’*. The relief of quitting the category of FGC is not surprising; in a recent Swedish study the majority of Swedish Somali men (92%) reported a preference for marrying someone without FGC, or with only a minor form such as pricking [46]. The women’s dissociation from FGC and desire to surgically manage and adapt their genitals thus indicates a change in perceptions of what constitutes the ideal female body.

Some women said that having undergone CR involved a change in how they perceived themselves. Patricia said: *‘It is among the best decisions I have made in my life*, *I have become a new person’*. This change was related to a sense of symbolically taking charge of their bodies. Several women talked about CR as an act of courage and expressed pride at being the first among their friends and family to take such a decision. Fanta talked about CR as an important statement: *‘I think I am the only one in my family who have made this move*. *That feels big’*. This is in line with what Weitz and Kwan’s view [47] that bodily choices are loaded with political meaning. Thus, CR was loaded with a sense of agency. CR then becomes a political statement, an act of ‘breaking free’ from the categorisation of ‘women with FGC’ and a kind of symbolic liberation which affected their relationships and overall lives.

### Reduced physical pain

While non-provoked physical pain had not been a primary reason for seeking out CR for most of the women, some described having experienced diminished physical discomfort, pain, or clitoral over-sensitivity after surgery. Leila said: *‘It is not as oversensitive as before*, *when it rubbed against my underwear’*. Others described that removal of the scar tissue had made the clitoris more soft and sensitive. For some, pain during sexual intercourse due to scar tissue, tightness and ingrown hairs had disappeared. This demonstrates that some women experienced physical improvements after CR.

### ‘Not only good’—Aesthetic, functional or process-related disappointment after CR

While none of the women regretted having CR, as at least some of their expectations of the surgery (see [28] for an overview of the motives and expectations of the surgery) had been met, some reported disappointment with certain aspects involved in the CR process. Their disappointment or resentment was largely related to access to, the process of, or the aesthetic or functional outcomes of the surgery.

While not a common complaint, some women reported difficulties in attending the accompanying psychosexual consultations due to not living in the Stockholm area or difficulty in gaining access to CR surgery because their gynaecologist refused to issue a referral. Behar had to visit a couple of gynaecologists before finding someone willing to issue a referral for CR specialist care: ‘*I really fought for the referral*, *I went to two gynaecologists before I succeeded*, *they didn’t want to send the referral*.*’* Gynaecologists’ reluctance to issue referrals for CR have been noted elsewhere [48], and seem to relate to a lack of knowledge about CR, but also to the limited evidence regarding the outcomes of CR surgery or to considering CR a scam. Villani [49] has noted that women requesting CR in France are required to display a certain image of ‘maturity’ in order to be considered eligible for surgery. This means that they have to produce a particular narrative about their motives and expectations in order to be considered eligible for CR. Only women who are able to convince their gynaecologists that they have the ‘right’ motivations and expectations, thus demonstrating their ‘maturity’, are allowed to undergo CR. While Behar had been persistent and fought to obtain access to CR, despite meeting two reluctant gynaecologists, she believed that such resistance could hinder other women from undergoing CR. Even if first-line doctors, including gynaecologists, have the intention to act appropriately and protect women from disappointment or even harm, blocking women from obtaining CR when they ask for it is paternalistic.

In Sweden, CR surgery involves obligatory psychosexual counselling, a model used in many countries [27]. This means that women are required to meet a psychosexual therapist at the hospital in order to be eligible for surgery. Here, they talk about their psychosexual and FGC-related problems, and fill in questionnaires about their sexual and psychological state. While some women found these consultations really meaningful, many criticized this part of the treatment and said it was exhaustive, time-consuming and psychologically draining. Soheila said:

*It is too much psychologically, I was totally exhausted when it was over, and that wasn’t the operation in itself (…) I don’t think, if you don’t really want the operation, then I don’t think you want to suffer through this psychological test. I think it was very exhausting, it is too much of this, but I understand if there are women who have had post-traumatic stress (…) But if you don’t have that then it is like this, I was at least very affected far after the operation was over, I was like ‘how glad I am that it is over’, all my energy was gone*.

Some said that they thought that the CR process, which often stretched over years, was a potential barrier for many women. They considered this unfair, and thought the process should be ‘fast and easy’; after all it was minor surgery. Most women had abandoned the psychosexual counselling as soon as they had been operated on.

The women’s negative view of the psychosexual counselling stands in stark contrast to the views of the experts in the area who promote psychosexual counselling as an important prerequisite and part of the CR treatment [16, 27, 49–51]. Psychosexual counselling is meant to distinguish those ‘ready’ for surgery from those who are not [49], make women think thoroughly about their decision, help them adapt to their new clitoris, and/or prevent negative outcomes such as PTSD [52]. Sharif Mohamed et al. [16] note that psychosexual therapy may also be an alternative to CR itself and a help to reduce misconceptions about sexual function. It is thus interesting that few of the women in this study saw much value in this aspect of the treatment. Upon hearing about the suggestion of offering cut women psychosexual counselling instead of the actual surgery as a solution to their problems, some got upset. Behar said: ‘*It is typical that when it is a woman’s issue*, *one recommends therapy*’. For her as well as others, it was precisely the physical part of CR that was important; because FGC involved having physical tissue removed, it was restoration on the level of the physical, not the psychological, that was important. Yet, even if emphasising the physical aspect of the surgery, some women appreciated the psychosexual counselling, saying that it had helped them to be more open and less shameful about their FGC. One woman interviewed for the pre-operative study [28] had abandoned the idea of the surgery because she felt helped just by talking about her problems (with the counsellor and others). This is also the driving idea behind advocates for psychosexual counselling prior to CR [16]. The reasons behind so many of the women’s rejection of the psychosexual counselling accompanying CR need further investigation.

Furthermore, some women expressed disappointment regarding their genital appearance after CR; they had hoped that their genitals would resemble uncut genitals to a larger degree than was the case. Some thought that their clitoris was ‘too small’ or that their genitals looked ‘strange’ after the surgery. Natalie was one of those who resented the size of her clitoris. She said: ‘*First*, *I was happy about how it looked*, *then I was disappointed because it was so small*.*’* The problem with the retraction of the clitoris after CR has been noted in other studies [20, 53]. While this could be related to misconceptions regarding the physical outcomes of the CR surgery as well as the natural size of the clitoris [54] it seems at present difficult for surgeons to guarantee avoidance of clitoral retraction.

Others talked about disappointment with their genitals still looking ‘too open’ due to a lack of labia. Leila, who had previously been infibulated, but surgically opened in early adolescence, was one of those whose primary motivation for CR had been to restore her genital appearance. Post-surgery she was disappointed, thinking that her genitals still looked ‘too open’. She said: ‘*I am not fully satisfied with the visual result; I would have wanted to restore the labia*.*’* While some surgeons have started to experiment with restoring labia by inserting fat tissue [26], this is still in an experimental phase, and the outcomes of such reconstruction need to be explored. Yet, most surgeons do not seem to be aware of the importance for cut women to have their genital appearance restored. This might sometimes involve labia restoration, especially in women with FGC Type III [55]. It could also be that surgeons are reluctant to restore the labia as they consider this, in contrast to CR, ‘unnecessary’ from a functional point of view, and that CR then becomes ‘aesthetic’ surgery. Another reason could be that it is a difficult procedure from a surgical point of view. Yet, because the genital appearance is important for women seeking CR, the possibility for labia reconstruction needs to be taken into consideration if one wants to help cut women.

At the same time, many women said they understood that they would not ‘look 100% uncut’. Thus, even if they ‘had hoped for more’ they were glad that something had been done and that their genitals looked better than before the CR. Furthermore, some of the women who had initially been dissatisfied with the aesthetic outcomes of the surgery, said that they had ‘calmed’ down after getting assurance from the surgeon at the post-operative check-up where they were able to compare pre- and post-operative genital pictures. Ami said:

*Ami: I told him that it [the clitoris] had gone back and like that, and that I thought that it would be another operation. But he looked and then he didn’t think it was necessary. He felt the clitoris then, and that it was in its place*.

*Interviewer: Ok*.

*Ami: And then I got to see a picture of myself and it looked really good after all, I thought*.

Some women reported other concerns of a more physical character. This involved experiencing that the clitoris was oversensitive after surgery, bordering on painful, even if this had been more present in the first months after the surgery. Ilham said: ‘*It was very oversensitive*, *I couldn’t touch down there*, *it is still very sensitive*.*’* Zara was another woman who had sought CR to reduce clitoral pain; she had lived with an oversensitive clitoris for as long as she could remember, something that had affected her life and relationships, particularly her sex life. Yet, she said that the surgery had not led to any improvements, and she was now awaiting another operation.

Other women had the opposite problem and were disappointed that their clitoris had not become *more* sensitive. Soheila said that her reconstructed clitoris felt unnatural and hard, even if softer than before the surgery: ‘*It feels a little fake*, *hard*, *although not as hard as before*.*’* Others mentioned that the ‘new’ clitoris was positioned in a different place than before, even if they were not necessarily bothered by this. One woman recounted that she now felt a spot next to the clitoris that was without any sensation. Yet, negative physical outcomes of CR were seldom mentioned, something which the literature supports; in a systematic review only 2.3% of women experienced a worsening in clitoral pleasure and 2.0% recounted genital pain that was not present before surgery [56]. Still, the possibility of a worsening of physical pain needs to be taken into consideration when discussing the potential risks involved in CR.

### Grateful for the recognition by the Swedish healthcare

Despite resentments and disappointment in regard of some aspects of CR, most women expressed gratitude to the Swedish healthcare for the possibility of undergoing CR surgery free of charge. This gave them a sense of having their FGC-related concerns taken seriously, which made them feel recognised as victims of an unjust and wrongful crime and in need of healthcare. Behar said: ‘*That someone takes care of you*, *wants to give you what you have lost*, *means a lot’*. And Patricia said: ‘*They did what they could and I am very proud of them and myself*. *I am very*, *very grateful’*. Several women talked about the liberation of having a choice of doing something about their FGC, which gave them a sense of power. Helen said: *‘The possibility of having a choice is very important*, *it feels like I can regain the power over my body’*.

Immigrant-specific healthcare needs are often neglected in European countries [17], something that may leave FGC-affected women’s concerns invisible. Ovesen [57] discusses the importance of institutional recognition, which is always unequal as existing social structures render some bodies more worthy of care and protection than others. Failed recognition may imply disavowal of existence which heightens bodily precariousness or ‘bioprecarity’ (see Griffin and Leibetseder [58] for more on ‘bioprecarity’). CR as a specialised healthcare service may not be relevant for the majority population in Sweden, but it can be seen as one way of responding to immigrant and cut women’s needs, and thus as a form of institutional recognition. Yet, Ovesen [57] argues, increased visibility of certain bodies is complex and can also lead to misrepresentation, attack and stigmatisation, which instead heightens bioprecarity. In the case of cut women, increased visibility as ‘victims of FGC’ might involve a (mis)representation of cut women as ‘damaged’. This self and other categorisation of ‘victims’ could be the very thing that prompts women to seek out CR. Yet, the construction of FGC as criminal and ‘wrong’ is already established in most parts of the world [14]. Thus, instead of blaming cut women for perceiving and experiencing their bodies according to this narrative, they deserve having their concerns taken seriously.

### Methodological considerations

This study was the second part of a larger study where both motivations and expectations of CR surgery were investigated [28]. While follow-up with all women included in the first part was an aim, not all women could be contacted for the follow-up interview. Whether this was due to their discontent with the surgery or for other reasons is difficult to say. We therefore included four additional interviews to provide a larger data set and because we identified their motives and expectations of the surgery as similar to those included in the first part.

The rapport established with the majority of the participants is likely to have made them feel more at ease during the interview situation. The study is qualitative and did not systematically measure sexual behaviour and pleasure before and after the surgery. Neither did we use a questionnaire such as the Female Sexual Function Index (FSFI). Instead the study provides a subjective account of the women’s reported differences in experience post-surgery regarding several aftereffects, including sexuality, aesthetics and pain. The data set of 18 interviews is large in the context of a follow-up study of women undergoing CR, as there is a dearth of research in this context. Yet, because the study is qualitative, we do not claim that our data is generalizable to a larger population. However, the insights into the nuanced aspects of benefits and disappointments after CR provide valuable information for healthcare providers meeting FGC-affected women, as well as researchers wanting to understand and explore issues related to CR further.

And while the majority of participants came from only one country (Somalia), this can be a strength as it provides representation of cut women in Sweden, and also differs from most other CR-related research where the participants mainly originate from West African countries.

One strength of this study is the interdisciplinary research team consisting of a surgeon specialised in CR and another genital surgeon (second author), professor in gender studies (third author), and healthcare scholars specialised in research on FGC and CR (first and last author). This helped to sharpen the methodology and interpretation of the data. And while the data collection and major analysis were undertaken by the first author, all authors were involved in discussing the findings and in the final analysis.

The analysis was undertaken to provide a nuanced account of the outcomes of the surgery as well as its benefits and disappointments. The variety of experiences detailed in the interviewees’ accounts reflects a complex picture of how women experience the process and outcome of CR surgery. To enable readers to judge our interpretation as well as its transferability to other contexts, we have included rich and illustrative quotes.

## Conclusion

This study has demonstrated that many women experience physical, sexual and psychosocial benefits from undergoing CR. They reported reduced genital pain, improvements in their sex lives, and a sense of feeling more empowered and at ease in their bodies. Yet, not all women reported such improvements; some expressed that they had ‘hoped for more’.

A primary motivation for CR for many women had been to improve their sex lives, which they understood as negatively affected by the FGC. The difference in their experience of post-surgery sex is interesting in this context. Although the current study did not systematically investigate pre- and post-operative clitoral sensation, it seems that many women experienced an improvement in clitoral sensation. Does that mean that the surgery’s effects in restoring sensitive clitoral tissue is not always successful? Or does it imply that some women such as Soheila did not explore the possibility of clitoral sex, but expected that vaginal/coital sex would also improve after CR? These are questions to be explored in future studies.

## Supporting information

S1 File Interview guide English. (DOCX)

S2 File Interview guide Swedish. (DOCX)
