# Supplementary material for: The benefits and disappointments following clitoral reconstruction after female genital cutting: A qualitative interview study from Sweden
Source: PLoS One. 2021 Jul 21;16(7):e0254855. doi: 10.1371/journal.pone.0254855 (PMC8294499; doi:10.1371/journal.pone.0254855)
Supplement: S1 File — (DOCX) [file pone.0254855.s001.docx]

**Topic guide - klitoris rekonstruktion studie**

**Intervjuguide – svenska**

**Pre-operative questions**

History of FGC

Talking about FGC (family, friends, healthcare personnel)

Sexuality (sexual function, masturbation, orgasm)

Relational factors (friends, family, previous/present partners)

Identity, sense of belonging, integration (body experience, self-confidence, tradition, modernity)

Physical, sexual and psychological experiences of living with FGC

Motivation for seeking out clitoral reconstruction (CR)

Expectations of CR (sexual, visual, body, psychological, emotional, identity related)

Experience with the healthcare (information, understanding, advice, referral, advice)

**Post-operative interview:**

Talking about FGC (family, friends, healthcare personnel)

Sexuality (sexual function, masturbation, orgasm)

Relational factors (friends, family, previous/present partners)

Identity, sense of belonging, integration (body experience, self-confidence, tradition, modernity)

Physical, sexual and psychological experiences of CR

Expectations met?

***Be om å få kontakta igen efter 1 år (telefonnummer och e-postadress)***
