# Supplementary material for: The benefits and disappointments following clitoral reconstruction after female genital cutting: A qualitative interview study from Sweden
Source: PLoS One. 2021 Jul 21;16(7):e0254855. doi: 10.1371/journal.pone.0254855 (PMC8294499; doi:10.1371/journal.pone.0254855)
Supplement: S2 File — (DOCX) [file pone.0254855.s002.docx]

**Ämnesområder - klitoris rekonstruktion studie**

**Pre-operativt intervju:**

KKS historia

Prata om KKS

*(familj, vänner, vårdpersonal)*

Sexualitet (*sexuell funktion, orgasm, masturbering)*

Relationsfaktorer *(venner, familj, nuvärande/tidligere partners)*

Identitet, känsla av tillhörighet, integration *(kroppsuppfattning/självförtroende, tradition, nya/gamla)*

Fysiska, sexuella, psykologiska erfarenheter av med att leva med KKS

Motivation för att efterfråga klitoris rekonstruktion

Förväntningar till KR (sexuella, estetiska, kropp, psykologiska, emotionella, idenitetsrelaterad)

Erfarenhet med vården

*(Information, förståelse, stöd, rådgivning, remiss, råd från helsopersonell )*

**Pre-operativt intervju:**

Prata om KKS

*(familj, vänner, vårdpersonal)*

Sexualitet (*sexuell funktion, orgasm, masturbering)*

Relationsfaktorer *(vänner, familj, nuvarande/tidigare partners)*

Identitet, känsla av tillhörighet, integration *(kroppsuppfattning/självförtroende, tradition, nya/gamla)*

Konsekvenser av KR

Infriade förväntningar?

***Be om å få kontakta igen efter 1 år (telefonnummer och e-postadress)***
